# Microstructure and Texture Evolution of Mg–Gd–Y–Zn–Zr Alloy by Compression–Torsion Deformation

**DOI:** 10.3390/ma12172773

**Published:** 2019-08-28

**Authors:** Ping Xu, Jianmin Yu, Zhimin Zhang

**Affiliations:** School of Materials Science and Engineering, North University of China, Taiyuan 030051, China

**Keywords:** Mg–Gd–Y–Zn–Zr alloy, compression–torsion, microstructure, texture

## Abstract

Mg–13Gd–4Y–2Zn–0.5Zr alloy was subjected to compression–torsion deformation at 450 °C with a strain rate of 0.001–0.5 s^−1^ using a Gleeble 3500 torsion unit. The effects of compression–torsion deformation on the microstructure and texture were studied, and the results showed that with the decrease of strain rate, the texture strength decreased, the number of dynamic precipitated particles increased, the degree of recrystallization increased, and the dynamic recrystallization mechanism changed from a continuous dynamic recrystallization mechanism to a continuous and discontinuous dynamic recrystallization mechanism. Along the direction of increasing radius, the degree of dynamic recrystallized grain (DRX) increased, the number of dynamic precipitated particles increased, and the texture strength slightly increased.

## 1. Introduction

With the increasing requirement of lightweight equipment in aerospace, military, transportation, and other fields, magnesium alloys have attracted more and more attention [1]. In recent years, it has been found that Mg–Gd–RE alloys with long period ordered stacking phase (LPSO) have attracted extensive attention because of their superior mechanical properties at room temperature and high temperature [2,3,4,5,6]. The thermoplasticity of magnesium alloy is poor due to the number of the slip system being limited by the hexagonal closed-packed structure of magnesium, which restricts the application of magnesium alloy to a great extent [7,8]. At present, the study of the plastic deformation mechanism and microstructure in rare earth magnesium alloys containing an LPSO phase by compression deformation is an important method to obtain the strengthening and toughness mechanism of rare earth magnesium alloys. Li et al. [9] reported that when the Mg–Gd–Y–Zr alloy was subjected to a compressive deformation with a strain of 0.7 at 350 °C, a large number of twins appeared in the coarse original grains due to the limited activation of the slip system. Xu et al. [10] reported that when the compression experiment of Mg–9Gd–2.9Y–1.9Zn–0.2Ca alloy with a strain of 0.9 was carried out at 350 °C, no twinning was observed due to the hindrance of a large number of lamellar LPSO phases. Zhang et al. [11] used the compression deformation of Mg_6_Gd_3_Y_1_Zn_0.4_Zr to study the influence of the LPSO phase with different morphologies on the dynamic recrystallized grain (DRX) process, and founded that the high-density lamellar LPSO phase hindered the opening of the base slip and the activation of the kink deformation, and at the same time hindered the DRX process. However, the block-shaped LPSO phase played an important role in promoting the crystallization process.

Hagihara et al. [12] confirmed that the (0001) [11,12,13,14,15,16,17,18,19,20] basal dislocation slip is the main deformation mode of the LPSO phase. Xu et al. [10] reported that when the loading direction of compression deformation was basically parallel to the base plane of the LPSO phase, the base slip was difficult to open due to having a low Schmid factor. At this time, the kink band of the LPSO phase is adapted to the deformation by the generation and the synchronous slip of the dislocation pairs of the opposite sign of the base plane. The opening of the deformation mechanism is not only related to the loading direction, but also significantly related to the temperature, strain variable, and strain rate. Li et al. [9] reported that a large number of twins appeared in the coarse original grains, and no dynamic recrystallized grains (DRXs) were founded due to the limited numbers of activation of the slip system in the compression deformation with a strain variable of 0.7 at 350 °C. Lv et al. [13] reported that when Mg–2.0Zn–0.3Zr–0.9Y alloy is subjected to compression deformation at 250 °C, DRXs nucleated at the original grain boundary when the strain rate was low, and DRX grain nucleated in the twins after increasing the strain rate.

Torsional deformation is a simple experimental deformation method that can obtain large strain without failure of specimen, and can investigate the microstructure evolution and mechanical properties of magnesium alloy under the condition of large plastic deformation [14,15,16]. Currently, there are not enough reports on the torsion deformation of magnesium alloys; existing research studies mainly focus on the torsion deformation of pure magnesium and AZ31. Chen et al. [17] studied the Swift effect on the tensile and compressive yield asymmetry of AZ31 magnesium alloy by torsional deformation. The results of Shu et al. [18] show that different torsional paths can improve the tensile and compressive asymmetry, and may enhance the aging strengthening effect of AZ91 alloy. At present, there are few studies on the torsional deformation behavior of rare earth magnesium alloys. Yu et al. [19] reported that the stress–strain curve of Mg–11.95Gd–4.5Y–2Zn–0.37Zr alloy under hot torsion is similar to the stress–strain curve under thermocompression. Thermal torsion significantly reduces the texture under different deformation conditions.

Using the composite experiment of compression and torsion, this paper studies the deformation behavior of rare earth magnesium alloy containing an LPSO phase under different stress states, which is an important experimental method for obtaining the strengthening and toughening mechanism of the rare-earth magnesium alloy. However, there is no research on rare earth magnesium alloys by deformation of the composite experiment of compression and torsion at home and abroad. This experiment is based on the Mg–13Gd–4Y–2Zn–0.5Zr (wt. %) alloy to investigate the evolution of microstructure and properties at different compression–torsion strain rates.

## 2. Materials and Methods

The Mg–13Gd–4Y–2Zn–0.5Zr alloy obtained by semi-continuous casting is used as the material for this experiment, and the size of the original bar is 330 mm × 1000 mm. The components obtained by ICP testing are shown in Table 1. The as-cast Mg–13Gd–4Y–2Zn–0.5Zr (wt. %) alloy was homogenized at 520 °C for 16 h and then cooled in air.

The torque and torsional angle should be controlled in a torsional test, and the compression displacement and axial load should be controlled in a compression test. Naturally, the relationship between the four deformation parameters needs to be considered in the compression–torsional process. In general, the relationship between shear stress and shear strain is used to explain the torsional experimental results in the torsional process. The strain of torsional process can be expressed by Equation (1):

(1) $$\tau =\frac{\sqrt{3}M(3+m+n)}{2\pi R_{0}^{3}}$$

The equivalent stress and equivalent strain during the compression–torsion process shall be calculated according to the following equation:

(2) $$\bar{\sigma_{eq}}=\sqrt{3\tau^{2}+\sigma^{2}}$$

Make:

(3) $$C=\frac{l_{1}^{3}}{l_{0}^{2}}-l_{0}+\frac{\theta^{2}R_{0}^{2}}{l_{0}}$$

(4) $$\varepsilon_{eq}=\frac{1}{2}\ln[\frac{l_{0}}{l_{1}}-\frac{1}{2l_{1}}(\sqrt{C^{2}+4\theta^{2}R_{0}^{2}}-C)]$$

where *M* is the torsional moment; *n* is the work hardening coefficient; *m* is the strain rate sensitivity coefficient; *R*_0_ is the radius of sample standard distance section (mm); *l*_0_ is the length of the sample distance section before deformation (mm); *l*_1_ is the length of the sample distance section after deformation (mm); *M* is the torsional radian (N·m); and *M*, *θ*, and *l*_1_ can be measured directly by a Gleeble 3500 torsion unit.

**Figure 1:**
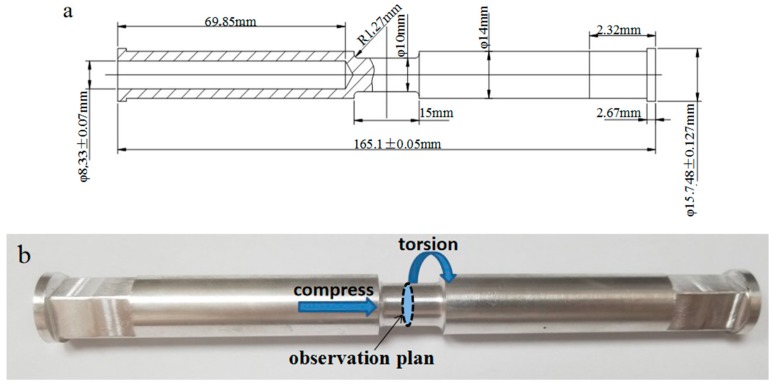

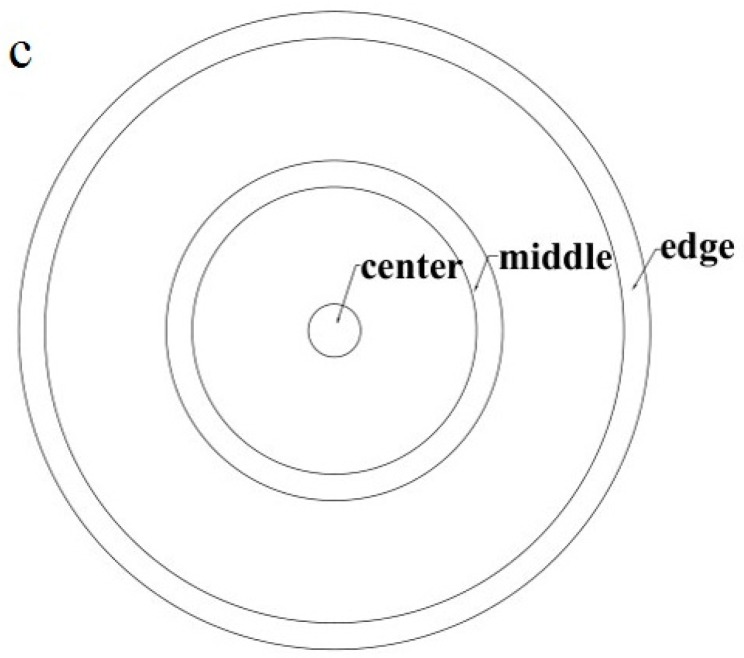
(**a**) The drawing of the compression–torsion specimen; (**b**) the pre-compression and torsional specimen; (**c**) the observation position of the specimen.

This experiment was carried out with the Gleeble 3500 axial large-load torsion test platform developed by DSI Company (New York, NY, USA) and the Precision Forming Center of North University of China. The compression–torsion specimen were sampled along the axial direction in the middle of the cast rod. In order to ensure that the sample is heated uniformly at a standard distance, the raw materials are specially processed into a specimen with hollow shoulders and an equal cross-sectional area at a standard distance, as shown in Figure 1a. The sample is held for 8 min after heating to the experimental temperature, and the force of 500 N is added before the experiment to ensure that the specimen is in contact with the indenter. The experiment was carried out at a temperature of 450 °C and a compression–torsion strain of 0.8 (axial compression of 5 mm and radial torsion of 0.5 turns) at the strain rates of 0.00 s^−1^, 0.01 s^−1^, 0.1 s^−1^, and 0.5 s^−1^, respectively. The specimen was cooled in the air when the compression–torsion tests were over. The middle of the cooled specimen was cut off by line cutting in the direction of the parallel end plane for performance testing. Due to the different cumulative torsion strains along the radius direction, to investigate the effect of different strains on the microstructure, the microstructure was observed in three different parts of the sampling plane: the center, the middle, and the edge. The width of each part is 0.5 mm, as shown in Figure 1c. The strains of the three regions were 0.4, 0.56, and 0.8, respectively. The samples were polished with water sandpaper to 5000 mesh and polished. Then, the samples were was corroded in corrosion solution made up of 1 g of picric acid, 2 mL of deionized water, 2 mL of glacial acetic acid, and 14 mL of anhydrous ethanol for 5 s, after which microstructure observation was carried out under a ZEISS-Image optical microscope (Oberkochen, Germany). A Hitachi SU5000 scanning electron microscope (Tokyo, Japan) was used to characterize the evolution of microstructure at high magnification. The polished samples were applied to EBSD (electron backscattered scattering detection) observation by using a voltage of 6.8 kV on the Laika ion beam thinner and thinning for 35 min. Data were processed using the commercial software package TSL OIM analysis 7.

## 3. Results and Discussion

### 3.1. Microstructure of the Alloy before Compression–Torsion Deformation

**Figure 2:**
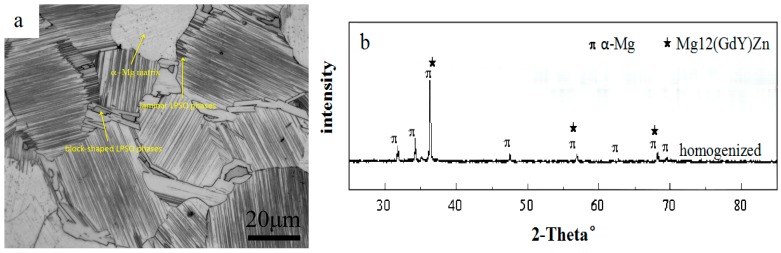
(**a**) The optical microscopy (OM, Zeiss, Jena, Germany) image after homogenization, and (**b**) the XRD spectrum after homogenization.

Figure 2 shows the microstructure and XRD (PANalytical, Almelo, The Netherlands) spectrum of Mg–13Gd–4Y–2Zn–0.5Zr (wt. %) alloy after homogenization. It can be seen from Figure 2a that the homogenized microstructure mainly contains an α-Mg matrix, lamellar LPSO phase distributed in an α-Mg matrix, and a block-shaped LPSO phase distributed at the grain boundary. The results of XRD in Figure 2b show that only the α-Mg matrix phase and Mg_12_(GdY)Zn phase are included in the homogenized microstructure. The average grain size after homogenization measured by the linear intercept method is 47.9 μm.

### 3.2. Evolution of Edge Microstructure at Different Strain Rates

#### 3.2.1. Observation of Microstructure

**Figure 3:**
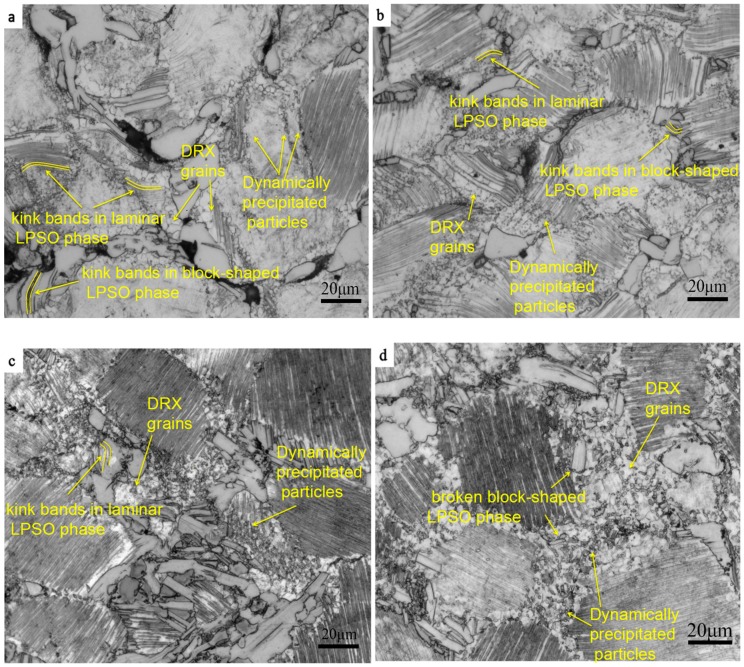
Microstructure of the edge region under different strain rates. (**a**) 0.5 s^−1^; (**b**) 0.1 s^−1^; (**c**) 0.01 s^−1^; and (**d**) 0.001 s^−1^.

Figure 3 shows the edge microstructure at different strain rates. As shown in Figure 3a, a large number of lamellar LPSO phases are kinked at the compression–torsion strain rate of 0.5 s^−1^. At the same time, the phenomenon of a kinked of block-shaped LPSO phase can be also observed, and a small amount of DRXs are formed along the interface between the block-shaped LPSO phase and the α-Mg matrix. At the same time, it is worth noting that a small number of fine precipitated phase particles precipitated at the grain boundary. It has been proven that the main deformation mode of the LPSO phase is the dislocation slip of the base plane in the direction of (0001) <11−20> [12]. When the loading direction is parallel to the base plane, the Schmid factor of the base slip is very small, which is basically negative. At this time, the slip of the base plane is difficult to open, and the kink bands are formed by the generation of dislocation pairs of opposite symbols on the base surface and synchronous slip to adapt to the stress concentration formed by the high strain rate at the grain boundary [12]. A large number of dislocations accumulate rapidly at the grain boundary at a high strain rate, which causes the local stress to concentrate and the deformation resistance to increase. In the initial stage of deformation, a large number of dislocations are accumulated in the kink bands first, rather than at the recovery stage and in the DRX. Small DRXs are formed along the interface between the LPSO phase and α-Mg matrix, and at the grain boundary of the α-Mg matrix with the further increase of strain. The formation of DRXs at the interface between the LPSO and the α-Mg matrix was also reported by Chen et al. [20]. DRXs are nucleated by consuming the high-density dislocations that accumulate at the grain boundary.

Figure 3b shows the microstructure with a strain rate of 0.1 s^−1^. It can be observed that the degree of crystallization in the microstructure is larger than that in the strain rate of 0.5 s^−1^. At this time, the phenomena of the more lamellar LPSO phase and less block-shaped LPSO phase being kinked can also be observed in the microstructure, which is because the strain rate is still high at this time, resulting in high deformation resistance. It can also be observed in Figure 3b that the number of small particles precipitated dynamically increases obviously and the distribution is more dispersed, but it mainly occurs around and inside the lamellar LPSO phase. Dislocation stacking and recombination have more sufficient time with the decrease of strain rate; the low-angle grain boundary was formed, and its subsequent further increase lead to the nucleation of subgrains. Then, the increase of misorientation leads to the formation of DRXs in further deformation. At the same time, it can be observed in Figure 3b that the DRXs had formed not only at the grain boundaries, but also in the grains containing the lamellar LPSO phase. This is mainly due to the hindrance of the dislocation movement of the lamellar LPSO phase. The dislocation density in this region increases rapidly by blocking the dislocation between layered LPSO phases, and the phenomenon of recrystallizing occurs due to reaching the energy needed for nucleation.

Figure 3c shows the microstructure when the deformation rate reaches 0.01 s^−1^. It can be seen that the kink bands of the lamellar LPSO phase have basically disappeared in the microstructure, but the slightly kinked phenomenon of the block-shaped LPSO phase can also be observed. At this time, a large number of small particles were precipitated under the action of heat, which were mainly distributed among the DRXs at the grain boundary, and their size had slightly increased. The degree of DRX in the microstructure is slightly higher than that in the strain rate of 0.1 s^−1^. More dislocations can be absorbed by the grain boundary by decreasing the deformation rate due to the recovery time of the deformation increase. There is also enough time for the nucleation and growth of DRXs.

When the strain rate further decreases to 0.001 s^−1^, as shown in Figure 3d, there is no kinked phenomenon in the microstructure, and there is a significant increasing trend in the size of the DRXs. It can be also observed that the block-shaped LPSO phase began to break up into small pieces, which are distributed among the DRXs. The number of dynamically precipitated small particles is larger, and the distribution is more diffuse. The shape of the precipitated phase is not only granular, but also acicular, and is mainly distributed among the DRXs. It indicated that the basal slip was easier to open in this deformation condition, and no kinks were needed to adapt to the deformation. Since the temperature of 450 °C belongs to the high temperature of magnesium alloy deformation, the atoms have high activation energy, and the migration speed of the grain boundary is also large in this temperature. There is enough time to make the DRXs grow when the strain rate is low.

**Figure 4:**
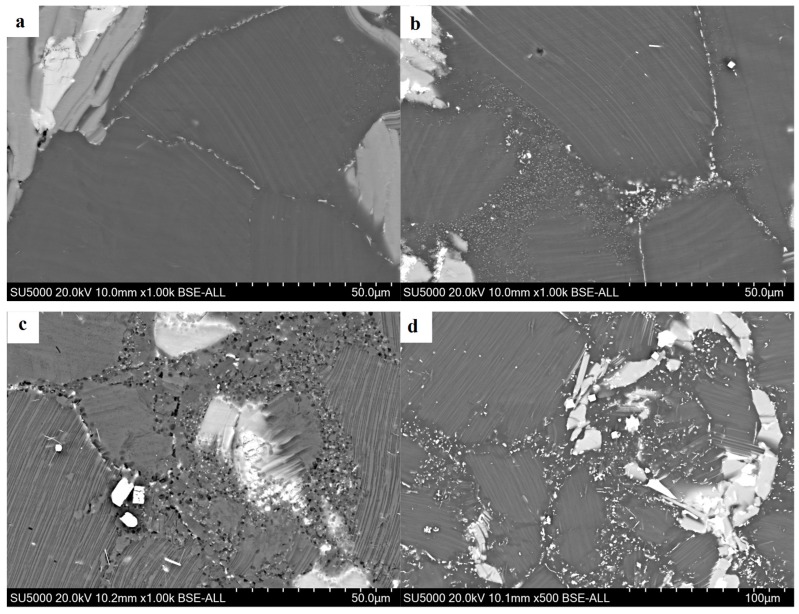
(**a**–**d**) Backscattered electron (BSE) image of strain rates of 0.5 s^−1^, 0.1 s^−1^, 0.01 s^−1^, and 0.001 s^−1^, respectively.

Figure 4 shows the backscattered electron (BSE) image of the edge regions at different strain rates. It can be seen that the number of dynamically precipitated particles exhibits an increasing trend with the decrease of strain rate, and their distribution developed from the grain boundary to among the DRXs, with a more diffuse distribution. Its morphology also developed from a single spheroidal shape to contain both spheroidal and acicular shapes. It can be seen that there are many more DRXs in the dynamic precipitation region than in the area without dynamic precipitation. Since the extensive lattice defects cause the original grain boundaries to have higher energy, the second phase particles are more likely to precipitate at the site. Furthermore, the dynamic precipitation process is a thermally active process; thus, sufficient time can not be provided to uniformly distribute dynamic precipitated particles under the conditions of this experiment. Some studies have shown that small particles larger than 1 μm can act as dynamic recrystallized nucleation points in the process of thermal deformation, which is called particle-stimulated nucleation (PSN) [21,22,23,24]. Ding et al. [25] reported that the second-phase particle obstructed the dislocation movement and caused the dislocation to accumulate around the second-phase particle that was precipitated dynamically in the process of continuous deformation [26]. When the dislocation density reached the critical value, the new grain may nucleate. Therefore, the degree of recrystallizing in the dynamic precipitated region is large.

#### 3.2.2. Dynamic Crystallization Mechanism

**Figure 5:**
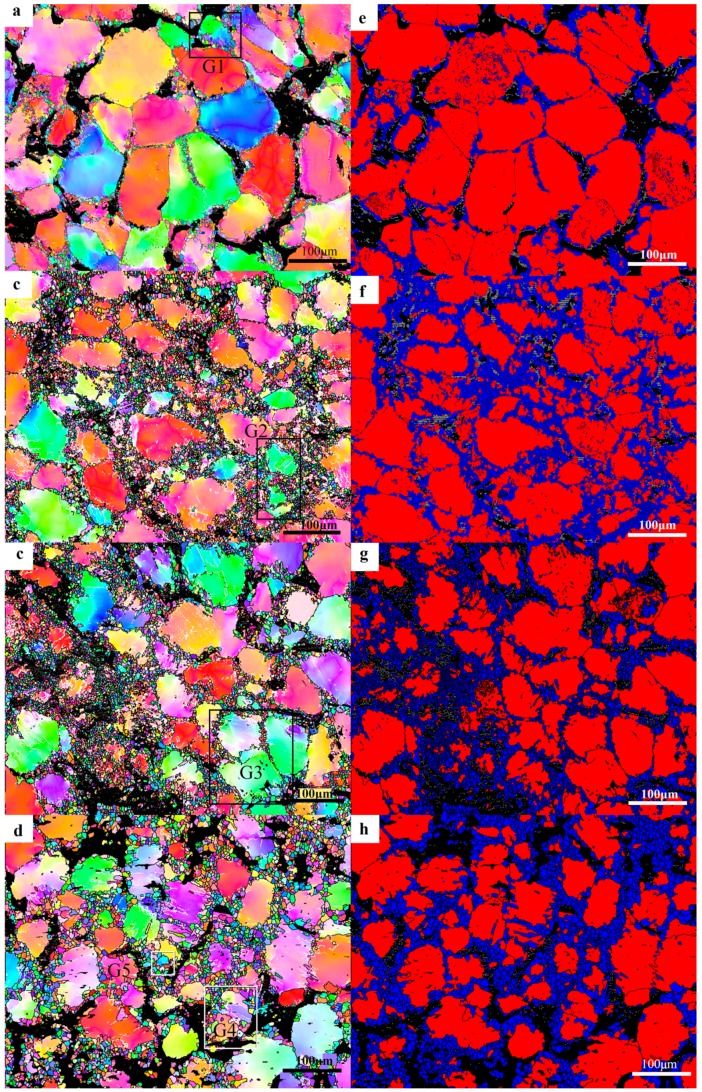
(**a**–**d**) are the inverse polar figure of strain rates 0.5–0.001 s^−1^ respectively, and (**e**–**h**) are the dynamic recrystallized grain (DRX) figures corresponding to (**a**–**d**), respectively.

It is easy to form stacking faults and extended dislocations because of the low stacking fault energy of magnesium, and it is difficult to cross-slip, so there is mainly dynamic recrystallization softening in the thermal deformation [27]. In order to reveal the grain dynamic crystallization mechanism under different deformation conditions in the process of compression–torsion deformation, the details of the edge microstructure under different deformation conditions at 450 °C were detected by the EBSD technique, and the results are shown in Figure 5. Figure 5a–d shows the inverse pole figure with strain rates of 0.5–0.001 s^−1^, respectively. The LPSO phase was marked black during data processing due to the lack of data of the LPSO phase in the EBSD database. It can be seen from Figure 5a that the degree of recrystallizing is small when the strain rate is 0.5 s^−1^. The degree of recrystallizing increases with the decrease of strain rate, and the grain size of DRX with the strain rate of 0.0001 s^−1^ is obviously higher than that under other conditions. Figure 5e–h show the DRX diagrams corresponding to a–d. According to the statistics of OIM 7.3 software, the DRX strain rates of 0.5 s^−1^, 0.1 s^−1^, 0.01 s^−1^, and 0.001 s^−1^ were 14.6%, 35.4%, 37.6%, and 41.3%, respectively. During this period, a large number of dislocations piled up at the grain boundary in an instant due to too little deformation time when the strain rate was 0.5 s^−1^, and the deformation energy was not released completely by DRX before the deformation process was over. When the deformation rate increased to 0.1 s^−1^, there was sufficient time to release the deformation energy, so the DRX rate was greatly improved. However, when the strain rate decreased from 0.1 s^−1^ to 0.001 s^−1^, the degree of DRX slowly increased from 35.4% to 41.3%, and the size of the DRXs had increased. There will be more sufficient time to consume the deformation energy by recrystallizing the nucleation at this stage with the decrease of the deformation rate. The dislocation accumulated at the grain boundary is limited, because the degree of deformation strain is consistent. That is to say, the decrease of the strain rate is beneficial to the nucleation of the DRX. The deformation time is prolonged due to the decrease of the deformation rate when the strain rate is 0.001 s^−1^. The DRXs grew up under the action of thermal energy at the strain rate of 0.001 s^−1^.

**Figure 6:**
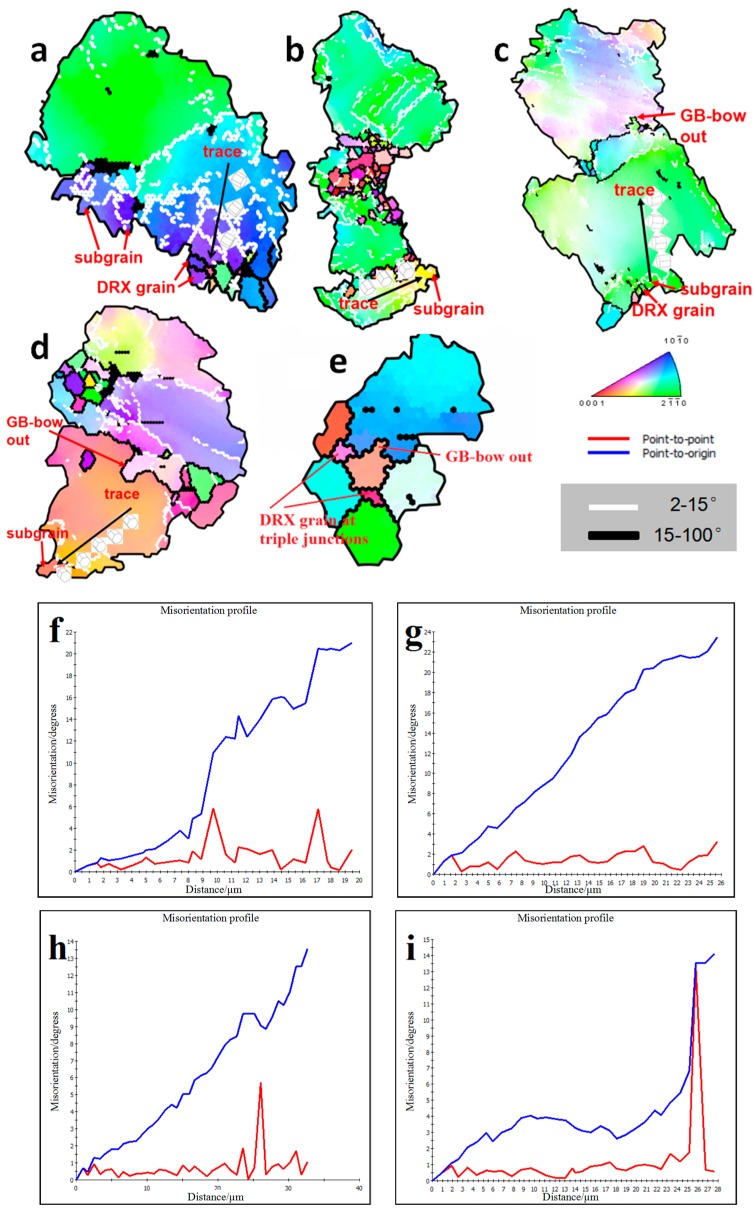
(**a**–**e**) An enlarged map of the selected area from Figure 5a–d; (**f**–**i**) the misorientation profiles measured along the trace in a–e, respectively.

In order to reveal the specific DRX mechanism of the edge microstructure under different deformation conditions, different small grains designated as G1–G5 were selected in Figure 5a–d for amplification study. Figure 6a–e shows enlarged images of the G1–G5 grains in Figure 5, respectively. Figure 6f–i show respectively the change of misorientation of trace in G1–G5. It can be seen from Figure 6f that the misorientation increases gradually with the increase of the distance from the origin. Subgrains are gradually formed at the grain boundaries with the increase of dislocation accumulation, and DRXs are finally formed with the further increase of misorientation. It can also be seen that the color of the DRXs is consistent with the original coarse grain, which is due to the continuous rotation of the subgrain leading to the formation of the high-angle grain boundaries (HAGBs). This process is an obvious continuous DRX (CDRX) process. The DRX mechanism at a strain rate of 0.1 s^−1^ was studied from G2, as shown in Figure 5b. It can be clearly seen that the deformation mechanism of DRX under this strain rate condition is still CDRX [10]. It can be seen from Figure 6h that the DRX mechanism at the strain rate of 0.01 s^−1^ is still the CDRX mechanism. However, the phenomenon of the large-angle grain boundary being arched out was observed in grain m, which indicates that in addition to CDRX, there is also discontinuity dynamic recrystallizing (DDRX) in the microstructure at this time. However, CDRX is still dominant at this time. It can be seen from Figure 6d,i that CDRX is the main mechanism of forming DRXs at the strain rate of 0.001 s^−1^. It is worth noting that the arch of the HAGBs is also seen in Figure 6e, and Sitdikov et al. [28] reported that the subgrain boundary caused by the interaction between the base slip and the non-base slip will cut off the original grain boundary and form DRXs. In addition, the grain magnification diagram of G5 in Figure 6e shows that small grains are formed at the triple junction of the DRXs, and the misorientation and color of the small grains is different from that of the surrounding coarse grains. These small grains are considered to be newly formed DRX, since they don’t contain the low-angle grain boundaries (LAGBs). Based on the above analysis, it can be determined that the DRXs formed by the previous CDRX continue to be further refined by the DDRX [29].

It can be seen from Figure 3 that DRXs occur mainly at the interface of the block-shaped LPSO phase and the α-Mg matrix, and there are few in the interfaces of the high-density layered LPSO phase. Through Figure 5e–h, it can be seen that the DRX are mainly nucleated at the interface between the α-Mg matrix and the block-shaped LPSO phase. The main reason for this phenomenon is that the block-shaped LPSO phase has higher yield strength than that of the α-Mg matrix [30]. It is easier to cause stress concentration caused by dislocation accumulation and rearrangement at the interface between the α-Mg matrix and block-shaped LPSO phase; then, the subgrain boundary is formed and gradually evolved into recrystallized grain. This is consistent with the reports of Zhang et al. [11], in which the high-density layered LPSO phase hindered the movement of basal slip to delay DRX, and the block-shaped LPSO phase had a promoting effect on DRX. It can also be seen from Figure 3d that the number of high-density lamellar LPSO phases increases with the decrease of the strain rate, which may be due to the lamellar LPSO phase being precipitated dynamically for a long period of time. The reason why there are more DRXs with a strain rate of 0.001 s^−1^ is that the dynamic precipitation of the lamellar LPSO phase has a limited effect on the DRX process [11].

### 3.3. Texture Evolution of Edge Part under Different Strain Rates

#### 3.3.1. Evolution of Texture

**Figure 7:**
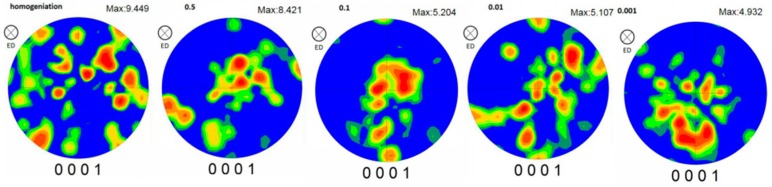
The (0001) pole figure at each strain rate.

Figure 7 is the (0001) pole figure of different strain rates. It can be seen that the compression–torsion deformation process has a weakening effect on texture, from 9.364 of the intensity of homogenization to 4.932 of the intensity of 0.001 s^−1^. It can also be seen that the texture intensity is gradually weakened with the decrease of the strain rate at the temperature of 450 °C.

**Figure 8:**
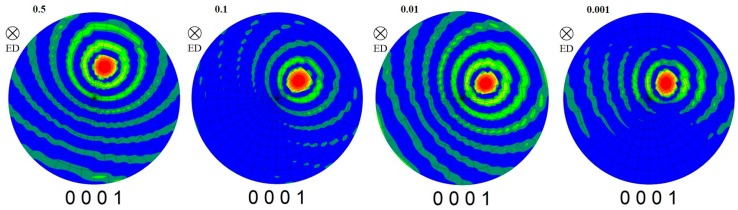
The (0001) pole figure of the maximum intensity position of the (0001) pole figure at each strain rate.

Figure 8 shows the (0001) pole figure of the position of maximum intensity selected from the pole figure at different strain rates. It can be seen that the position of the maximum intensity at each strain rate gradually developed from the random distribution of the homogenized to the direction of 45° deviation from the compression direction. This development indicates that the grain arrangement of the base plane changed from the random arrangement of the homogenization to the direction of 45°, and then to the compression direction.

#### 3.3.2. Effect of Recrystallizing on Texture

**Figure 9:**
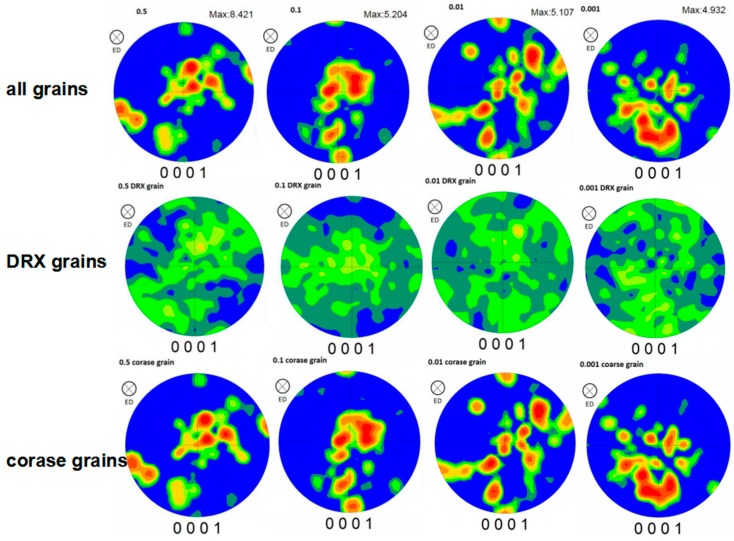
The (0001) pole figure of all the grains, DRXs, and coarse grains at different strain rates, respectively.

Figure 9 shows the (0001) pole figure of all the grains, DRXs and coarse grains at different strain rates. It can be seen that the weakening of the compression–torsion texture is mainly related to the degree of DRX. The compression–torsion texture is mainly contributed by coarse grains, because their pole figures are basically consistent. The influence of strain rate on texture is mainly reflected in the strain rate having a slight influence on the deformation temperature, and the higher strain rate can cause the local temperature to rise. Xu et al. [10] reported that the compressive strain rate of Mg–9Gd–2.9Y–1.9Zn–0.4Zr–0.2Ca alloy was carried out 10 s^−1^, which would lead to an adiabatic shear band due to the local temperature rise caused by the high strain rate, and DRX would occur near the shear band. However, this is mainly affected by the lower temperature, and the increase of deformation rate at a lower temperature will lead to a significant temperature rise. At the same time, the effect of deformation rate on texture is more complex when the temperature is high, mainly considering the texture changes caused by dislocation slip, grain rotation, grain boundary, and the intracrystal diffusion and dynamic crystallization caused by rate change.

### 3.4. Evolution of Gradient Microstructure in Compression–Torsion Deformation

#### 3.4.1. Microstructure

**Figure 10:**
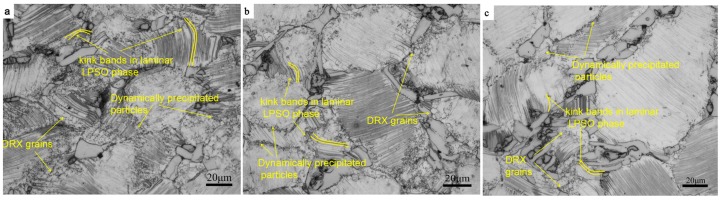
Microstructure of different regions at a strain rate of 0.1 s^−1^: (**a**) edge part; (**b**) middle part; (**c**) center part.

The deformed microstructure of compression–torsion is a typical gradient microstructure due to the torsional strain increases along the direction of radius increase. The change of gradient microstructure can well explain the evolution of microstructure with strain in the process of compression–torsion, because the law of increasing along the radius under different strain rate conditions is similar. The evolution of the microstructure along the radial direction is analyzed by taking the compression–torsion deformation of strain rate 0.1 s^−1^ as an example. Figure 10 shows the microstructure at different positions along the radius direction at the strain rate of 0.1 s^−1^. It can be seen that a large number of lamellar LPSO phases in the edge region are kinked, and the degree of crystallization is large. There is a large amount of dislocation accumulation and a large local stress concentration at the grain boundary due to the large strain in the edge region, at the strain rate. A large number of lamellar LPSO phases has kinked in order to adapt to the deformation. Meanwhile, the DRX occurred in this region in order to release stress and eliminate dislocation and the subgrain boundary.

It can be seen from Figure 10b that the number of kinked, DRXs, and dynamically precipitated particles show a significant decrease tendency compared with the microstructure at the edge. As shown in Figure 10c, the degree and quantity of kink bands are obviously lower than those in the middle part. It can be seen that only a minority of the DRXs appear at the interface between the block-shaped LPSO phase and the α-Mg matrix. This is mainly because the torsional strain in this region is too small; only the accumulation of dislocation at the LPSO phase interface is large, and the recrystalline grains are easy to grow there. 

**Figure 11:**
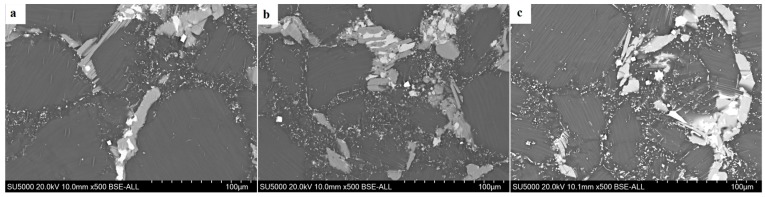
(**a**–**c**) BSE images of the center, middle, and edge of the strain rate of 0.001 s^−1^, respectively.

In order to clearly see the law by which the number of small particles precipitated dynamically with the increase of the radius, the compression–torsion microstructure with a strain rate of 0.001 s^−1^ was selected for observation. Figure 11 shows the BSE images of different regions with a strain rate of 0.001 s^−1^. It can be seen from the diagram that the number of dynamically precipitated phases also increases along the direction of radius increase.

**Figure 12:**
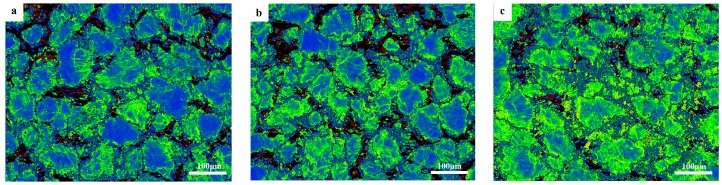
(**a**–**c**) KAM (Kernel Average Misorientation) maps of the side, middle, and heart parts at a strain rate of 0.001 s^−1^, respectively.

In a word, when the strain rate is 0.1 s^−1^, the DRX degree increases along the direction of radius increase from the center, and the number of kink bands of the lamellar LPSO phase and the number of dynamic precipitated particles also show the same trend. The main reason for this phenomenon is that the strain of torsional deformation in the radius direction is proportional to the radius, resulting in different local internal stresses in different regions of the microstructure. Figure 12 shows KAM (Kernel Average Misorientation) maps of different regions at the strain rate of 0.1 s^−1^. It can be seen that the residual stress in the microstructure increases along the direction of the radius increase. Due to the serious stress concentration, the number and degree of twisting of the lamellar LPSO phase increases, and the dislocation movement has a greater driving force, so that the local dislocation density reaches the critical recrystallizing density, which leads to the occurrence of recrystallizing.

#### 3.4.2. Dynamic Crystallization Mechanism

**Figure 13:**
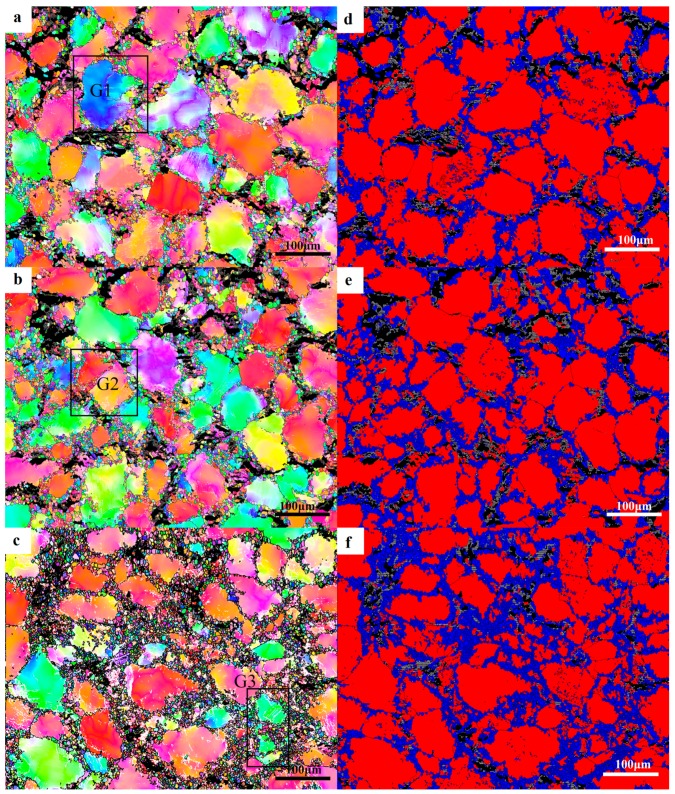
(**a**–**c**) The reverse pole figure of the center, middle, and edge of the strain rate of 0.1 s^−1^, respectively. (**d**–**f**) The corresponding recrystallizing diagrams, respectively.

The details of different regions of the strain rate of 0.1 s^−1^ at 450 °C were detected by the EBSD technique to reveal the grain refinement mechanism of different regions of the strain rate of 0.1 s^−1^ in the deformation of compression–torsion, and the results are shown in Figure 13. Figure 13a–c are the inverse polar figures of different regions at the strain rate of 0.1 s^−1^, respectively. Figure 13d–f are the recrystallized maps corresponding to different regions of the strain rate of 0.1 s^−1^. From Figure 13d–f, we can see that the number of DRXs increases along the direction of radius increase from the center. The degree of recrystallizing in the center, middle, and edge regions is 21.4%, 25.5%, and 32.8%, respectively from the statistics of recrystallizing diagrams in different regions. 

**Figure 14:**
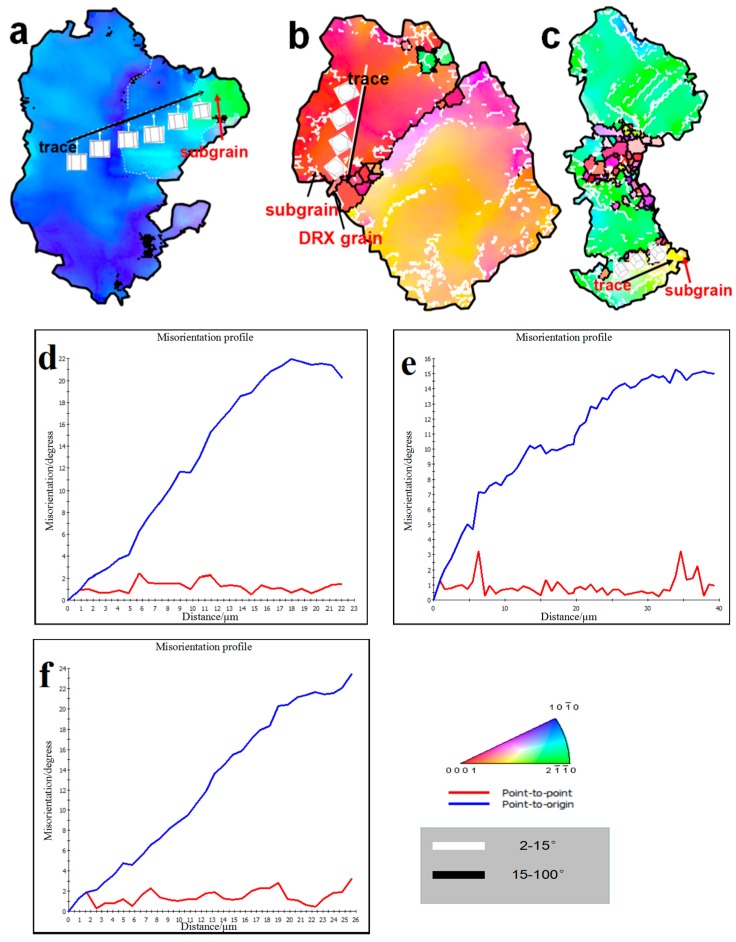
(**a**–**c**) An enlarged map of the selected area from Figure 13a–c; (**d**–**f**) The misorientation profiles measured along the trace in Figure 14a–c, respectively.

Figure 14a–c shows the grains selected from Figure 13a–c, respectively. Figure 14d–f shows the misorientation profiles measured along the trace in Figure 14a–c, respectively. It can be seen from Figure 14d–f that the misorientation increases gradually along the selected traces. At the same time, it can be also seen that the crystal in Figure 14a–c is rotated gradually along the trace, which is a typical CDRX characteristic. Therefore, when the strain rate is 0.1 s^−1^, the types of recrystallizing at different parts are CDRX.

#### 3.4.3. Texture Evolution of Gradient Microstructure

**Figure 15:**
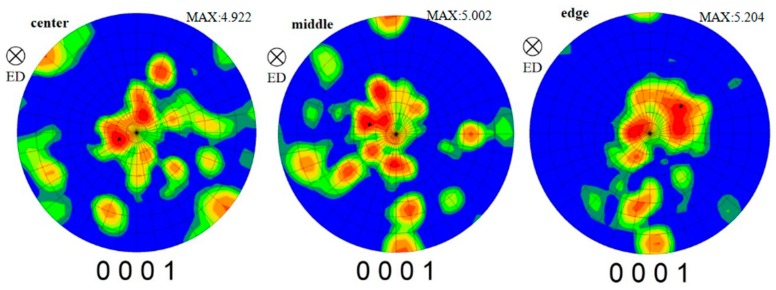
The (0001) pole figure of different parts under a strain rate of 0.1 s^−1^.

Figure 15 shows the (0001) pole figure of different regions at a strain rate of 0.1 s^−1^. Through the processing of the data, the strongest position of the pole figure was obtained, and is marked with a black pentagonal star in Figure 15. It can be seen that the maximum density tends to increase slightly along the direction of radius increase. The angle that indicates the strongest position of the pole figure deviates from the compression direction and increases gradually with the increase of radius. The deviation from the compression direction of the center, middle, and edge is about 20°, 30°, and 40°, respectively. This is mainly related to the difference of the strain contributions by torsion in different positions.

## 4. Conclusions

- The number of kink bands in the microstructure is gradually reduced with the decrease of the strain rate, and basically disappears when the strain rate is 0.01 s^−1^. The number of dynamic precipitated particles increased with the decrease of strain rate; its distribution is more uniform, and its morphology develops from a single sphere to a state of spheroidal and acicular coexistence. The number of dynamic precipitated particles and the number of kink bands increase in the direction of the increasing radius.
- The number of DRXs increased gradually with the decrease of the strain rate; the number of DRXs increased greatly from 0.5 s^−1^ to 0.1 s^−1^, which increased little between the strain rates 0.1–0.001 s^−1^. The type of DRX mechanism changed from CDRX to DDRX, and the DRXs grew under the effect of thermal activation energy at the strain rate of 0.001 s^−1^. As the strain increases along the direction of radius increase, the number of DRXs in the microstructure increased along the direction of increasing radius. The DRX types along the radius increase are all CDRX types when the strain rate was 0.1 s^−1^.
- The compression–torsion deformation will weaken the texture, and the degree of texture weakening was increased with the decrease of strain rate. The position of the maximum pole intensity and the compression direction deflected by about 45° at each strain rate. The strength texture was mainly contributed by the undeformed coarse grains. The texture increased weakly along the direction of the radius increase, and the angle deviating from the compression direction increased.

## Figures and Tables

**Table 1:** Composition of Mg–13Gd–4Y–2Zn–0.5Zr (wt. %).

Mg	Gd	Y	Zn	Zr	Si	Cu	Fe
Balance	12.88	4.00	2.00	0.50	<0.01	<0.01	<0.01
